# Pelvic floor symptoms and quality of life 1 year postpartum in Swedish primiparous women—A follow‐up of a randomized controlled trial

**DOI:** 10.1111/aogs.70216

**Published:** 2026-04-24

**Authors:** Malin Edqvist, Cecilia Häggsgård, Pia Teleman, Gunilla Tegerstedt, Gunilla Ajne, Susann Ullén, Helena Tern, Karin Ängeby, Hannah G. Dahlen, Christine Rubertsson

**Affiliations:** ^1^ Department of Women's and Children's Health Karolinska Institutet Stockholm Sweden; ^2^ Department of Women's Health and Health Professions Karolinska University Hospital Stockholm Sweden; ^3^ Department of Health Sciences, Medical Faculty Lund University Lund Sweden; ^4^ Department of Obstetrics and Gynecology Skane University Hospital Malmö Sweden; ^5^ Department of Clinical Science, Intervention and Technology (CLINTEC) Karolinska Institutet Stockholm Sweden; ^6^ University and Clinical Studies Sweden, Forum South Skane University Hospital Lund Sweden; ^7^ Centre for Clinical Research Karlstad Sweden; ^8^ Department of Health Science, Faculty of Health, Science, and Technology Karlstad University Karlstad Sweden; ^9^ School of Nursing and Midwifery Western Sydney University Sydney Australia

**Keywords:** episiotomy, OASI, pelvic floor dysfunction, quality of life, second‐degree tears

## Abstract

**Introduction:**

Long‐term pelvic floor symptoms after childbirth may impair women's quality of life. The aim of this study was to assess the prevalence of self‐reported pelvic floor symptoms 1 year postpartum in primiparous women by degree of perineal tear, with a focus on minor and major second‐degree tears, and their association with quality of life.

**Material and Methods:**

This prospective cohort study was based on data collected within a randomized controlled trial (the Oneplus trial). Women in the trial who had a vaginal birth and responded to a 1‐year postpartum follow‐up questionnaire were included. Data were collected between January 2020 and May 2021. The main outcome measures were pelvic floor symptoms assessed using the Pelvic Floor Distress Inventory (PFDI‐20), the Pelvic Floor Impact Questionnaire (PFIQ‐7), and study‐specific items related to suturing and perceived body image. Associations between type of perineal tear and pelvic floor symptoms and their impact on quality of life were examined using generalized linear models, estimating adjusted risk ratios (aRRs) with 95% confidence intervals (CIs). Trial registration: ClinicalTrials.gov, NCT03770962.

**Results:**

The cohort consisted of 1911 primiparous women. Among the tear categories investigated, major second‐degree tears were the most common (30.4%), followed by minor second‐degree tears (18.4%), episiotomy (9.8%), and obstetric anal sphincter injury (OASI) (5.3%). PFD symptoms were reported by 31.4–51.5% of the women. Women with OASI had an increased risk of colorectal–anal distress compared with those with no tear or a first‐degree tear (aRR 1.56, 95% CI 1.24–1.96). No associations were observed between minor or major second‐degree tears and pelvic floor symptoms. Increasing tear severity was associated with a higher likelihood of perceiving the vagina as narrow. No differences between tear categories were observed regarding impact on quality of life. Episiotomy was associated with a negative body image related to vaginal symptoms (aRR 1.45, 95% CI 1.03–1.99).

**Conclusions:**

Pelvic floor symptoms and their impact on quality of life were common 1 year postpartum, irrespective of perineal tear category. Minor and major second‐degree tears were not associated with an increased risk of pelvic floor dysfunction or reduced quality of life.

## Graphical abstract

In this follow‐up of a randomized controlled trial including 1911 primiparous women with a vaginal birth, minor and major second‐degree tears were not associated with pelvic floor dysfunction 1 year postpartum. OASI was associated with colorectal–anal symptoms, whereas episiotomy was associated with vaginal symptoms affecting body image.

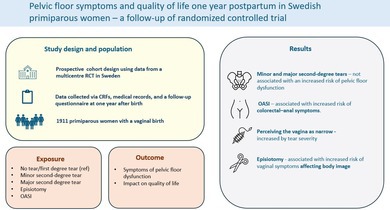

**Abbreviations**
aRR: adjusted relative risk
BMI: body mass index
CI: confidence interval
CRF: case report form
OASI: obstetric anal sphincter injury
PFD: pelvic floor dysfunction
RR: relative risk

**Key message**

Pelvic floor symptoms among primiparous women with a vaginal birth were common 1 year postpartum, regardless of tear severity. Minor and major second‐degree tears were not associated with pelvic floor dysfunction.

## 1 INTRODUCTION

Perineal injury following childbirth is common, particularly after the first vaginal birth.^1^, ^2^ In recent decades, increasing attention has been directed toward both the immediate and long‐term sequelae of childbirth‐related trauma.^3^ These long‐term outcomes include symptoms of pelvic organ prolapse (POP), fecal incontinence, and urinary incontinence, all of which may substantially impair women's physical functioning, psychological well‐being, and overall quality of life.^4^ Reported prevalences of pelvic floor symptoms vary across countries and subpopulations, partly due to differences in study methods, outcome definitions, and timing of postpartum assessment.^5^

Of the different types of tears, injuries to the anal sphincter (OASI) are among the most significant, as they contribute to short‐ and long‐term morbidity and are strongly associated with anal incontinence in childbearing women.^6^ Considerably less is known about the impact of second‐degree tears on long‐term pelvic floor dysfunction (PFD). This is despite the fact that second‐degree tears can include complex injuries with disruption of multiple perineal muscles and connective tissues, including structures that contribute to the perineal body and deeper vaginal support structures.^7^ However, morbidity associated with second‐degree tears and their relationship with PFD show conflicting findings.^8^, ^9^ Furthermore, qualitative data reveal that experiencing PFD symptoms during the first year after birth affects women both physically and emotionally, highlighting concerns about both current and future health.^10^, ^11^ Altogether, there remains insufficient knowledge regarding the impact of minor and major second‐degree tears on women's health, particularly with respect to perceived pelvic floor disorder symptoms and quality of life.

To investigate PFD symptoms and their impact on quality of life in relation to perineal trauma, this study used follow‐up data from a multicenter randomized controlled trial conducted in Sweden and designed to evaluate the effectiveness of a midwifery intervention to reduce OASI.^1^ The aim of the present study was to assess the prevalence of self‐reported PFD symptoms 1 year postpartum in primiparous women according to the degree of perineal trauma, with particular attention to minor and major second‐degree tears, and to investigate the association between PFD symptoms and quality of life.

## 2 MATERIAL AND METHODS

This study was conducted as a prospective cohort study based on data collected within the Oneplus multicenter randomized controlled trial.^1^ Data used for the present study include data from the 1‐year follow‐up questionnaire and data from case report forms (CRFs) completed by the midwives after each birth and from the obstetric units' local databases (Obstetrix Cerner or Cosmic Cambio) (Table S1).

The Oneplus trial was designed to evaluate the effect of collegial midwifery assistance during the late second stage of labor, defined as two midwives attending the birth, on the incidence of obstetric anal sphincter injury (OASI) as the primary outcome and other degrees of perineal trauma as secondary outcomes. The trial was conducted across five obstetric units in Sweden, and the trial design allowed for sub‐classification of second‐degree tears into minor and major tears. No power calculation was performed for outcomes related to PFD or health‐related quality of life at 1 year postpartum. The full details of the Oneplus trial have been described in detail elsewhere.^1^ Data collection for the 1‐year follow‐up questionnaire took place between January 2020 and May 2021.

Inclusion criteria for participation in the 1‐year follow‐up questionnaire were participation in the trial (according to the original trial inclusion criteria: women aged 18–47 years who were either pregnant with their first child or planning a first vaginal birth after caesarean section, at ≥37 + 0 weeks' gestation, with a singleton live fetus in vertex presentation) and proficiency in Swedish or English.

In Sweden, it is standard practice to offer all women who have experienced vaginal birth a visual and digital vaginal and rectal examination after birth to assess and classify any perineal tearing. Following placental delivery and after informed consent has been obtained, the midwife or physician performs the initial examination. Swedish midwives and obstetricians receive training in the assessment of perineal tears and suturing during their pre‐registration education, and all hospitals provide additional training, often on an annual basis.^12^ Midwives classify and suture first‐ and second‐degree tears and vaginal tears, whereas obstetricians classify and suture complicated vaginal and perineal injuries, including OASI. If an episiotomy is considered, a mediolateral or lateral episiotomy is recommended.^13^

Before trial commencement, standardized educational sessions were provided to the midwives at each participating site. These sessions included detailed training on pelvic floor anatomy and the classification of perineal trauma, with particular emphasis on the identification and classification of anatomical structures involved in second‐degree tears. To ensure accurate classification of tears, the study protocol specified that the primary midwife examine the woman together with an independent assessor (midwife or obstetrician).^14^ Data from the CRFs included questions on tear classification and the anatomical structures involved. Tear categories included intact perineum (no tear), first‐degree tear, second‐degree tear, and vaginal tears. A minor second‐degree tear was defined as involving only the m. bulbocavernosus, whereas a major second‐degree tear included a tear involving both m. bulbocavernosus and m. transversus perinei, and if the assessors reported a tear where the sphincter was visualized but not torn.

### 2.1 Exposures

The exposure was categorized as no tear or first‐degree tear (reference category), minor second‐degree tear, major second‐degree tear, episiotomy, and OASI. Data on OASI were retrieved from medical records using ICD codes O70.2 or O70.3. Data on first‐degree tears, minor and major second‐degree tears were obtained from the CRFs. Data on episiotomy was retrieved from the local databases, cross‐checked, and merged with episiotomies reported in the CRFs (Table S1). All episiotomies in the study were defined as mediolateral, with 13 episiotomies extending to an OASI. These were consequently categorized as such. Only 39 women had an isolated vaginal tear, and it was therefore decided not to use this variable as an exposure due to the limited number of cases. Perineal tears were exclusively categorized into a single category meaning that those involving several anatomical structures, such as OASI, were solely categorized within the OASI group.^12^

### 2.2 Outcomes

Outcomes related to PFD were obtained from the 1‐year follow‐up questionnaire, which covered maternal background and topics related to the first year after birth, such as breastfeeding, sexuality, and mental health. The outcomes of interest were pelvic floor symptoms and their impact on quality of life, assessed using the Pelvic Floor Distress Inventory (PFDI‐20), the Pelvic Floor Impact Questionnaire (PFIQ‐7),^4^ and additional study‐specific questions (Tables S2 and S3).

The PFDI‐20 comprises three subscales (POPDI, CRADI, and UDI). For all items, women are first asked whether each statement applies (*yes/no*). If *yes*, they are asked to rate the level of bother on a four‐point Likert scale ranging from *“not at all”* to *“quite a bit”*. Additional study‐specific items with the same response options, related to POP symptoms and/or suturing, were added, including: *“Do you think that your vaginal opening is too wide?” and “Do you think that your vaginal opening is too narrow*?” The PFDI‐20 is commonly scored by calculating total scores for each subscale.^4^ In the present study, we chose to analyze symptom presence rather than symptom severity; the analysis was therefore based on the initial yes/no responses, categorized as “*no symptoms*” (0) or “*symptoms*” (1). In addition to POPDI, the POPDI item addressing vaginal bulge symptoms: “*Do you usually have a bulge or something falling out that you can see or feel in your vaginal area?*” was analyzed as a single item, as this symptom has been shown to be indicative of POP symptoms.^15^

PFIQ‐7 comprises three subscales (POPIQ, CRAIQ, and UIQ). Women are asked to rate the extent to which symptoms affect their daily functioning, social well‐being, and mental health during the past 3 months using a four‐point Likert scale ranging from 0 (*“not at all”*), 1 (*“somewhat”*), 2 (*“moderately”*), and 3 (*“quite a bit”*). The research team further added one item assessing body image: *“My symptoms lead to negative thoughts and feelings about my body.”* The PFIQ‐7 is commonly scored by calculating subscale scores, with higher scores indicating greater impact on quality of life.^4^ In the present study, we chose to analyze impact as present or absent rather than by severity; responses were therefore dichotomized, with 0 indicating “*no impact*” and 1–3 indicating “*any impact*”.

Following consultation with women who pilot‐tested the questionnaire, items on urinary and fecal incontinence were answered only by women reporting these symptoms, to reduce respondent burden. To further shorten the questionnaire, and in consultation with the urogynecologists in the research group, the UDI was reduced to three items and CRADI was reduced to six items (Table S3). As symptoms of prolapse and their association with second‐degree tears are less well studied, all women were asked to complete all POPDI and PFIQ items related to POP.

In addition to the PFDI‐20 and PFIQ‐7, self‐reported urinary incontinence, involuntary passage of gas, and fecal incontinence were analyzed. Urinary incontinence was assessed using the item “*Do you suffer from urinary incontinence?*” with response options yes, sometimes, or no, and was dichotomized as incontinence (yes/sometimes) or no incontinence. Involuntary passage of gas and fecal incontinence were assessed using yes/no items (Table S2).

### 2.3 Covariates

All covariates were defined a priori based on existing evidence and clinical relevance. The selected covariates included maternal age, body mass index (BMI), mode of birth, birthweight, and ethnicity, all of which have previously been associated with perineal trauma and PFD.^5^, ^8^, ^9^, ^16^

### 2.4 Statistical analysis

Continuous variables were summarized as means and standard deviations or medians with interquartile ranges, and categorical variables were presented as numbers and percentages. Associations between type of perineal tear and pelvic floor symptoms and impact on quality of life at 1 year postpartum were examined using generalized linear models with a log link, first unadjusted and then adjusted for maternal age, body mass index (BMI), birthweight as continuous variables, and mode of birth and ethnicity as categorical variables. Results are presented as relative risk estimates comparing each tear category with the reference category no tear/first‐degree tear, together with 95% confidence intervals (CI). Analyses were conducted using complete‐case analysis. All analyses were performed using R (version 4.4.3; R Core Team 2025).

## 3 RESULTS

**FIGURE 1:**
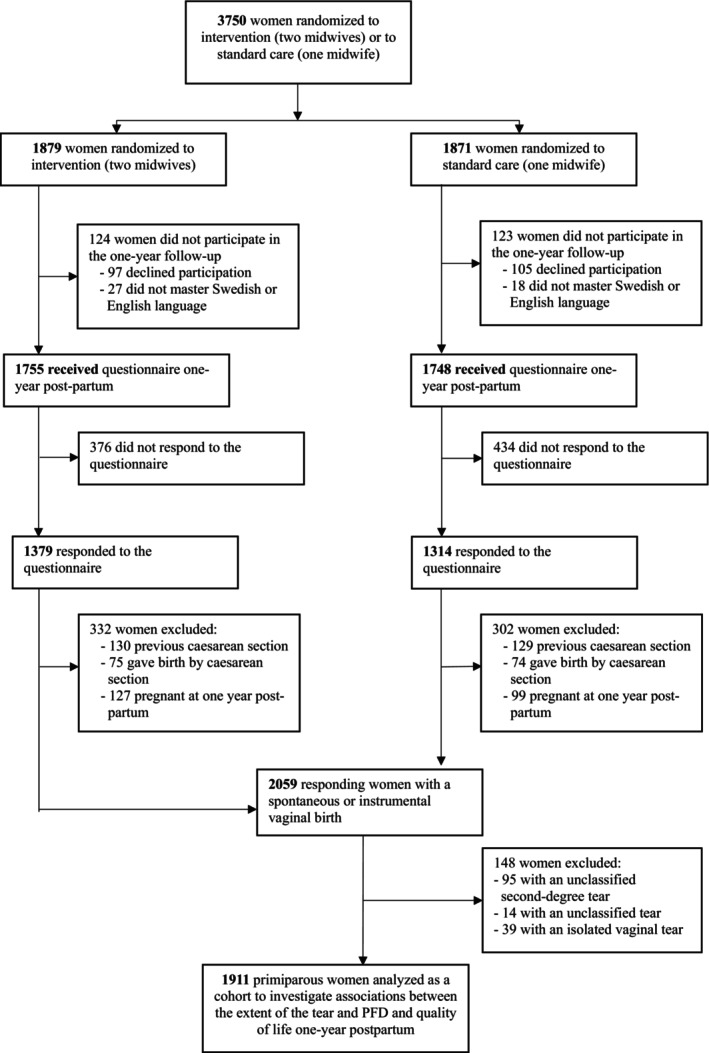
Flow diagram of women included in the Oneplus trial, participation in the 1‐year follow‐up questionnaire, and the final study cohort.

The original trial included 3750 women, of which 3503 were proficient in Swedish or English and consented to participate in the follow‐up questionnaire at 1‐year postpartum. Of these, 2693 women responded to the questionnaire 1 year after birth, yielding a response rate of 76.9%. For this study, we excluded those with a previous caesarean section, those who gave birth by caesarean section, or were pregnant at the 1‐year follow‐up, which resulted in 2059 women with a spontaneous or instrumental vaginal birth remaining for analysis (Figure 1). During the analysis, an additional 148 women were excluded: 53 with an unclassified tear or isolated vaginal tear, and 95 with an unclassified second‐degree tear. Thus, the final cohort included 1911 women (Figure 1).

Table 1 presents maternal background and labor characteristics stratified by tear category. Maternal age ranged from 29.8 to 30.7 years. Across all groups, the mean maternal BMI ranged from 24.0 to 24.7, falling within the normal‐weight category, and most respondents had completed at least 3 years of university education. Regarding mode of birth, 84.6% of the 1911 women had a spontaneous vaginal birth and 15.4% an instrumental birth. Among women who sustained an OASI, 34.3% had an instrumental birth compared with 6.1% of those with no tear or a first‐degree tear (Table 2). Similarly, women with OASI had the highest mean birthweight (3586 g, SD 416), whereas the lowest mean birthweight was observed among women with no tear or a first‐degree tear (3427 g, SD 418) (Table 1).

**TABLE 1:** Background and labor and birth characteristics of the 1911 participating women.

	No tear/ first‐degree tear *n* = 691	Minor second‐degree tear *n* = 351	Major second‐degree tear *n* = 580	Episiotomy *n* = 187	OASI *n* = 102
	*n* (%)	*n* (%)	*n* (%)	*n* (%)	*n* (%)
Mean maternal age at birth (SD)	29.8 (4.29)	29.9 (4.16)	30.4 (4.14)	30.5 (3.77)	30.7 (4.13)
Mean BMI^a^ (SD)	24.0 (4.46)	24.0 (4.68)	24.7 (4.59)	24.2 (4.29)	24.6 (4.29)
Missing data	23 (3.3)	8 (2.3)	21 (3.6)	4 (2.1)	5 (4.9)
Maternal chronic disease^b^	84 (12.2)	44 (12.5)	78 (13.4)	29 (15.5)	17 (16.7)
Tobacco use (smoking)	30 (4.3)	15 (4.3)	15 (2.6)	7 (3.7)	2 (2.0)
Missing data	1 (0.1)	1 (0.3)	1 (0.2)	1 (0.5)	0 (0.0)
Educational level					
Compulsory school	17 (2.5)	4 (1.1)	3 (0.5)	1 (0.5)	1 (1.0)
Upper secondary school	164 (23.7)	63 (17.9)	115 (19.8)	31 (16.6)	20 (19.6)
University 1–3 years	160 (23.2)	92 (26.2)	131 (22.6)	52 (27.8)	23 (22.5)
University >3 years	322 (46.6)	181 (51.6)	311 (53.6)	91 (48.7)	52 (51.0)
Other^c^	27 (3.9)	10 (2.8)	19 (3.3)	12 (6.4)	4 (3.9)
Missing data	1 (0.1)	1 (0.3)	1 (0.2)	0 (0.0)	2 (2.0)
Ethnicity					
Nordic	542 (78.4)	267 (76.1)	460 (79.3)	143 (76.5)	80 (78.4)
European	70 (10.1)	29 (8.3)	49 (8.4)	16 (8.6)	8 (7.8)
African	12 (1.7)	3 (0.9)	12 (2.1)	7 (3.7)	2 (2.0)
Middle Eastern	29 (4.2)	22 (6.3)	27 (4.7)	8 (4.3)	5 (4.9)
South American	10 (1.4)	6 (1.7)	6 (1.0)	2 (1.1)	1 (1.0)
Asian	24 (3.5)	22 (6.3)	20 (3.4)	10 (5.3)	5 (4.9)
Missing data	4 (0.6)	2 (0.6)	6 (1.0)	1 (0.5)	1 (1.0)
Onset of labor					
Spontaneous	522 (75.5)	258 (73.5)	402 (69.3)	141 (75.4)	78 (76.5)
Induction	169 (24.5)	93 (26.5)	178 (30.7)	46 (24.6)	24 (23.5)
Mode of birth					
Spontaneous vaginal birth	649 (93.9)	309 (88.0)	494 (85.2)	117 (62.6)	67 (65.7)
Instrumental birth*	42 (6.1)	42 (12.0)	86 (14.8)	70 (37.4)	35 (34.3)
Second stage of labor—minutes (median, IQR)	94 (53.75–157.5)	107 (60.75–172.5)	124 (68–201.25)	124 (76–204.0)	131 (76–187.5)
Missing data	1 (0.1)	1 (0.3)	2 (0.3)	0 (0.0)	0 (0.0)
Birth weight (mean, SD)	3427 (418)	3503 (430)	3590 (436)	3575 (409)	3586 (416)

* Instrumental birth includes vacuum extraction and forceps; forceps comprised five cases.

^a^ Body Mass Index kg/m^2^.

^b^ Composite variable including diabetes, chronic hypertension, asthma/pulmonary diseases, heart disease, epilepsy, endocrine diseases, chronic kidney diseases, Crohn's disease, ulcerative colitis, and systemiclupus erythematosus.

^c^ Other includes post‐secondary education of less than 3 years.

**TABLE 2:** Types of perineal trauma and assessment of tears among the 1911 participating women.

*N* = 1911	*n* (%)
Perineal trauma	
No tear	184 (9.6)
First‐degree tear	507 (26.5)
Minor second‐degree tear	351 (18.4)
Major second‐degree tear	580 (30.4)
Episiotomy	187 (9.8)
OASI	102 (5.3)
Third‐degree tear	94 (4.9)
Fourth‐degree tear	8 (0.4)
Tear assessed by two assessors	1606 (84.0)
Missing data	11 (0.6)
Rectal examination	1817 (95.1)
Missing data	50 (2.6)

Second‐degree tears were the most common type of tear (49.8%) and among subclassified second‐degree tears, major second‐degree tears were more frequent (30.4%) than minor second‐degree tears (18.4%) (Table 2). The prevalence of episiotomy was 9.8% overall (Table 2), 7.2% among women with a spontaneous vaginal birth, and 25.5% among those with an instrumental birth.

Urinary incontinence was a prevalent symptom, reported by 37.6%–42.0% of the women (Table 3). When assessed by the UDI, no significant differences were reported between the tear categories regarding symptoms of urinary distress (Table 4).

**TABLE 3:** Prevalence of self‐reported incontinence among the 1911 women responding to the 1‐year follow‐up questionnaire.

	No tear/first‐degree tear *n* = 691	Minor second‐degree *n* = 351	Major second‐degree tear *n* = 580	Episiotomy *n* = 187	OASI *n* = 102
	*n* (%)	*n* (%)	*n* (%)	*n* (%)	*n* (%)
Urinary incontinence^a^	267 (38.7)	147 (42.0)	238 (41.2)	70 (37.6)	39 (39.0)
Missing data	1 (0.1)	1 (0.3)	2 (0.3)	1 (0.5)	2 (2.0)
Flatus incontinence	105 (15.2)	52 (14.9)	96 (16.6)	23 (12.3)	32 (31.4)
Missing data	2 (0.3)	3 (0.9)	2 (0.3)	0 (0.0)	0 (0.0)
Fecal incontinence	17 (2.5)	18 (5.2)	20 (3.5)	9 (4.8)	8 (7.8)
Missing data	4 (0.6)	3 (0.9)	2 (0.3)	0 (0.0)	0 (0.0)

^a^ Include the response options “yes” and “sometimes”.

**TABLE 4:** Self‐reported pelvic floor symptoms among the 1911 women responding to the 1‐year follow‐up questionnaire, based on the pelvic floor disability index (PFDI‐20).

	No tear/first degree tear *n* = 691	aRR	Minor second‐ degree tear *n* = 351	aRR	Major second‐ degree *n* = 580	aRR	Episiotomy *n* = 187	aRR	OASI *n* = 102	aRR (95% CI)
	*n* (%)		*n* (%)		*n* (%)		*n* (%)		*n* (%)	
Symptoms of pelvic organ prolapse (POPDI)^a^	296 (43.2)	Ref (1.0)	155 (44.9)	1.07 (0.92–1.24)	264 (46.1)	1.06 (0.93–1.21)	90 (48.4)	1.16 (0.96–1.37)	52 (51.5)	1.17 (0.92–1.44)
Symptoms of colorectal‐anal distress (CRADI)^b^	215 (31.4)	Ref (1.0)	109 (31.6)	1.00 (0.82–1.21)	178 (31.2)	0.95 (0.80–1.13)	57 (30.8)	0.97 (0.75–1.24)	52 (51.0)	1.56 (1.24–1.96)
	Val									
Symptoms of urinary distress (UDI)^c^	235 (34.3)	Ref (1.0)	137 (39.5)	1.14 (0.96–1.34)	214 (37.2)	1.04 (0.89–1.21)	65 (35.3)	1.01 (0.79–1.26)	35 (34.7)	0.96 (0.70–1.26)
Single items										
Do you think that your vaginal opening is too wide?	76 (11.0)	Ref (1.0)	63 (18.1)	1.61 (1.17; 2.20)	101 (17.5)	1.49 (1.12–2.00)	45 (24.1)	2.25 (1.58–3.16)	13 (12.7)	1.27 (0.69–2.12)
Do you think that your vaginal opening is too narrow?	38 (5.5)	Ref (1.0)	34 (9.7)	1.93 (1.17–3.18)	77 (13.3)	2.94 (1.96–4.54)	16 (8.6)	1.67 (0.84–3.10)	16 (15.7)	3.43 (1.80–6.19)

*Note*: Percentages are based on valid responses.

Abbreviation: aRR: adjusted relative risk. Adjusted for: maternal age, BMI, mode of birth, birthweight, and ethnicity. The difference between the unadjusted and adjusted risk ratios was marginal.

^a^ Pelvic organ prolapse distress inventory (POPDI‐6). Missing data *n* = 21 (1.1%).

^b^ The scale was shortened and includes six items from the Colorectal‐Anal distress inventory (CRAD‐8). “*Do you feel you need to strain too hard to have a bowel movement?”, “Do you feel you have not completely emptied your bowels at the end of a bowel movement?”, “Do you usually lose stool beyond your control if your stool is well formed?”, “Do you usually lose stool beyond your control if your stool is loose?”, “Do you usually lose gas from the rectum beyond your control?”, “Do you experience a strong sense of urgency and have to rush to the bathroom to have abowel movement?”*. Missing data *n* = 23 (1.2%).

^c^ The scale was shortened and includes three items from the Urinary distress inventory (UDI‐6). *“Do you usually experience urine leakage associated with a feeling of urgency, that is, a strong sensation of needing to go to the bathroom?”, “Do you usually experience urine leakage related to coughing, sneezing or laughing?”, “Do you usually experience small amounts of urine leakage (that is, drops)?”* Missing data *n* = 17 (0.9%).

Fecal incontinence was reported by 5.2% of women with a minor second‐degree tear, 3.5% and 4.8% of those with a major second‐degree tear and episiotomy, respectively, and in 7.8% of women with an OASI (Table 3). Four women with major second‐degree tears, episiotomy, or OASI reported fecal incontinence requiring daily use of protective pads; no such reports were observed among women with no tear/first‐degree tear or minor second‐degree tear. Women with OASI had an increased risk of reporting colorectal–anal distress compared with women with no tear or a first‐degree tear (aRR 1.56, 95% CI 1.24–1.96), whereas no increased risk was observed for minor or major second‐degree tears or episiotomy (Table 4).

When POP symptoms were assessed using the POPDI, the prevalence of any symptoms ranged from 43.2.% in women with no tear or a first‐degree tear to 51.5% in women with OASI, with no statistically significant difference compared to the reference group (aRR 1.17, 95% CI 0.92–1.44). This was similar for minor second‐degree tears, major second‐degree tears, and episiotomy, where the prevalence of any bother ranged from 44.9% to 48.4% with no statistically significant differences compared with women with no tear or a first‐degree tear (Table 4). The prevalence of vaginal bulge symptoms ranged from 5.9% to 13.7% across tear categories, with the highest proportion observed among women with minor second‐degree tears (aRR 1.81, 95% CI 1.19–2.74).

Perceiving the vagina as too narrow was more common among women with minor second‐degree tears (aRR 1.93, 95% CI 1.17–3.18) and major second‐degree tears (aRR 2.94, 95% CI 1.96–4.54), compared with the reference group. This risk was further increased among women who sustained an OASI (aRR 3.43, 95% CI 1.80–6.19). Conversely, women with an episiotomy had the highest adjusted risk of perceiving the vaginal opening as too wide (aRR 2.25, 95% CI 1.58–3.16) when compared with women with the reference group: no tear or a first‐degree tear (Table 4).

The proportion of women reporting any impact on quality of life as assessed by the POPIQ‐7 ranged from 20.3% to 27.1% (Table 5). However, there were no statistically significant differences between the tear categories compared with the reference group. Additionally, women with an episiotomy more often reported a negative body image related to vaginal symptoms compared with the reference group (aRR 1.45, 95% CI 1.03–1.99) (Table 5). For urinary distress, the corresponding proportion of women reporting any impact on quality of life ranged from 18.4% to 23.2%, with no significant differences between the tear categories and the reference group. Furthermore, the low proportion of women reporting any impact of colorectal‐anal symptoms on quality of life precluded calculation of risk ratios (Table 5).

**TABLE 5:** Quality of life impact of pelvic floor symptoms among the 1911 women in the cohort assessed by pelvic floor impact questionnaire 7 (PFIQ‐7) and two additional items.

	No tear/first‐degree tear *n* = 691	aRR	Minor second‐degree *n* = 351	aRR	Major second‐degree *n* = 580	aRR	Episiotomy *n* = 187	aRR	OASI *n* = 102	aRR
	*n* (%)		*n* (%)		*n* (%)		*n* (%)		*n* (%)	
Any impact on QoL POPIQ‐7	128 (20.3)	Ref (1.0)	70 (22.7)	1.15 (0.88–1.50)	122 (24.4)	1.25 (0.99–1.57)	39 (23.1)	1.22 (0.86–1.68)	26 (27.1)	1.37 (0.90–1.97)
Any impact on QoL CRAIQ‐7	17 (2.5)	N/A	11 (3.2)	N/A	17 (2.9)	N/A	8 (4.3)	N/A	3 (2.9)	N/A
Any impact on QoL (UIQ‐7)	124 (18.6)	Ref (1.0)	68 (20.5)	1.13 (0.85–1.47)	125 (22.6)	1.14 (0.90–1.44)	33 (18.4)	0.96 (0.66–1.36)	23 (23.2)	1.17 (0.75–1.71)
Single items										
Vaginal symptoms—affecting body image^a^	116 (16.8)	Ref (1.0)	70 (20.1)	1.22 (0.92–1.60)	117 (20.2)	1.21 (0.95–1.55)	44 (23.5)	1.45 (1.03–1.99)	21 (20.6)	1.23 (0.76–1.88)
Urinary symptoms—affecting body image^b^	83 (12.0)	Ref (1.0)	41 (11.8)	0.90 (0.65–1.23)	82 (14.2)	1.02 (0.78–1.33)	23 (12.4)	0.95 (0.62–1.40)	10 (9.8)	0.77 (0.40–1.30)

*Note*: Percentages are based on valid responses. POPIQ‐7: Pelvic Organ Prolapse Impact Questionnaire. Missing data *n* = 207 (10.8%). CRAIQ‐7: Colorectal‐Anal Impact Questionnaire. Missing data *n* = 9 (0.5%). UIQ‐7: Urinary Impact Questionnaire. Missing data *n* = 82 (4.3%).

Abbreviation: aRR: adjusted relative risk. Adjusted for: maternal age, BMI, ethnicity, mode of birth and birthweight.

^a^ *“My vaginal symptoms/difficulties lead to me having negative thoughts and feelings about my body”*. Missing data *n* = 6 (0.3%).

^b^ *“My bladder control symptoms/difficulties lead to me having negative thoughts and feelings about my body”*. Missing data *n* = 8 (0.4%).

## 4 DISCUSSION

In this cohort of 1911 primiparous women, pelvic floor symptoms were common 1 year postpartum, regardless of perineal tear severity. The prevalence of urinary incontinence in our cohort was comparable to that reported in a recent Swedish study.^17^ In contrast to the findings of Huber et al., who observed an association between second‐degree tears and stress urinary incontinence, we found no evidence that any specific tear category was associated with increased urinary distress.^8^ Our findings are further consistent with those reported by Gommesen et al., in which no elevated risk was identified for individual tear categories.^18^ In contrast to urinary incontinence, the prevalence of fecal incontinence in our cohort was lower than that reported in previous studies,^8^, ^19^ even among women with OASI.^8^, ^20^ The elevated risk between OASI and colorectal‐anal symptoms is well established and supported by previous literature.^6^ Nevertheless, fecal incontinence was observed across all tear categories. The continence mechanism is complex, and several factors may contribute to the development of fecal incontinence following childbirth, including trauma to the pelvic nerves, endopelvic fascia, and pelvic floor muscles, as well as individual phenotype.^6^

The prevalence of POP symptoms assessed using the POPDI ranged from 42.8% to 51.0%, which is higher than previously reported estimates of approximately 13% at 3 months and 10% at 3–5 years postpartum in studies using POPDI.^21^, ^22^ Since pelvic floor symptoms in our study were dichotomized to experiencing symptoms or not, this may explain the higher prevalence observed. However, the prevalence of POP symptoms is known to vary widely (2.2–45%), as shown in a recent systematic review.^23^ Since POPDI is a patient‐reported outcome measure, it captures perceived symptom burden and women may have clinically demonstrable prolapse without significant symptoms.^24^ We further observed an association between vaginal bulge symptoms and minor second‐degree tears. This contrasts with previous studies reporting no association between second‐degree tears and POP symptoms.^8^, ^9^ The study by Macedo et al. may, however, have been limited by sample size, particularly within subgroups of second‐degree tears.^9^ As this is an observational finding, causality cannot be inferred, and the association may reflect other underlying factors not investigated in the present study, such as levator ani muscle injury. However, levator injuries have previously been shown to be primarily associated with OASI.^25^ Moreover, although the item concerning vaginal bulge symptoms has been shown to be indicative of POP symptoms,^15^ it has, to our knowledge, not been validated for the population investigated in this study. This finding should therefore be interpreted with caution, and future research is needed using questionnaires specifically developed and validated to capture symptoms and problems reported by women in relation to childbirth.^26^

Women with minor second‐degree tears more frequently reported that the vaginal opening felt too narrow. For this outcome, which likely reflects the effect of suturing,^11^ the association was stronger with increasing tear severity. More extensive perineal tearing may increase the risk of using too much suture material and/or tightening the perineal muscles too much. In contrast, experiencing the vagina as too wide was more common among women with episiotomy. Since episiotomy has been associated with infection and wound complications,^27^, ^28^ an impaired healing may contribute to this symptom.

In this study, approximately 20 percent of women reporting urinary incontinence and POP symptoms also reported a negative impact on quality of life. Urinary and fecal incontinence have previously been shown to be associated with impaired quality of life,^29^, ^30^ supporting the burden and relevance of these symptoms for women. However, when the different tear categories were compared with the reference category, no differences in quality of life impact were observed. Interestingly, women with an episiotomy had an increased risk of reporting a negative body image in our study. Results from a Swedish register‐based study showed an association between episiotomy and dyspareunia.^31^ Although dyspareunia was not assessed in the present study, we previously analyzed dyspareunia at 30–60 days postpartum.^12^ At that time point, few women had resumed sexual intercourse, and no association with episiotomy was observed. Negative thoughts and feelings related to the body may reflect an interplay between physical symptoms and emotional or experiential factors. Furthermore, this finding may be related to women's experience of being subjected to the intervention, as episiotomy has been associated with experiences of obstetric violence, which in turn have been linked to postpartum depression and post‐traumatic stress disorder.^32^ Quality of life is a multidimensional construct encompassing physical and mental components, influenced by factors such as depressive symptoms, partner support, body satisfaction, and parental satisfaction.^33^ These factors may partly explain why the presence of symptoms does not uniformly translate into impaired quality of life across injury categories.

As pelvic floor symptoms and their impact on quality of life were common 1 year postpartum, irrespective of perineal tear severity, clinical follow‐up during the first year after birth needs to place greater emphasis on women's reported symptoms. Although follow‐up has traditionally focused on women with OASI, our results indicate that perceived symptoms are also common in other groups. Accessible and tailored postpartum care may therefore help address care needs among a broader group of women during the first year after birth.^10^

A major strength of this study is the relatively large cohort of primiparous women. The rigorous trial design, high inclusion rate, and detailed study protocol, which enabled subclassification of second‐degree tears, further strengthen the study. In addition, all midwives received comprehensive and standardized training, with particular emphasis on the classification of second‐degree tears. The use of two assessors in most cases, together with the high proportion of rectal examinations performed to determine tear extent, enhances the accuracy of the classification. Furthermore, the high response rate indicates the importance of this topic to women and strengthens the overall validity of the findings. Finally, the use of validated patient‐reported outcome and experience measures represents an additional strength.

Several limitations should be acknowledged. First, symptoms were self‐reported, and for pelvic floor disorders, particularly POP symptoms, perceived symptoms may differ from those identified through clinical assessment.^24^ Second, the sample size calculation was based on the trial's primary outcome and was not intended to detect differences in specific PFD symptoms or their impact on quality of life. Although nearly 2000 women were included, the study may nevertheless have been underpowered, particularly with respect to less prevalent symptoms such as fecal incontinence. Third, the choice of instruments and how they were used can be considered a limitation. Although the PFDI‐20 and PFIQ‐7 are validated in the Swedish context,^33^ they were designed to evaluate the effect of interventions and treatment effects and are most commonly applied in older populations and for women with anatomical signs of PFD.^34^ However, they have been used in similar settings and populations.^21^, ^22^ Moreover, PFDI‐20 and PFIQ‐7 are conventionally summarized as continuous scores derived from the individual item responses, providing overall measures of symptom burden and impact on quality of life.^33^ It should also be noted that we did not use a validated scale to assess body image. Validated scales assessing body image in relation to pelvic floor disorders exist; however, these have only been validated for pelvic organ prolapse.^35^ In contrast, validated scales that measure body image in relation to other pelvic floor outcomes, such as urinary and anal incontinence, are lacking.^35^ Future research should focus on developing instruments that capture body image in relation to the full spectrum of pelvic floor outcomes.

Additionally, in this study, we chose to dichotomize item responses into symptom versus no symptom and impact versus no impact on quality of life. The deviation from the original scoring may limit the comparability with other studies. However, the approach facilitates interpretation, a challenge that has been considered in research related to patient‐reported outcome measures.^36^ Finally, the generalizability of the findings is limited to primiparous women and similar care settings.

## 5 CONCLUSION

In this cohort of primiparous women, pelvic floor symptoms were common 1 year postpartum, and 20–30% of women reported an impact on quality of life, irrespective of perineal tear category. Minor and major second‐degree tears were not associated with an increased risk of overall pelvic floor dysfunction, whereas women with OASI had an increased risk of colorectal–anal symptoms. Increasing tear severity was further associated with a higher risk of perceiving the vagina as narrow, while episiotomy was associated with vaginal symptoms affecting body image. Given the high prevalence of symptoms and the substantial perceived impact on quality of life, ensuring accessible care during the first year after birth is important to support women experiencing pelvic floor symptoms.

## AUTHOR CONTRIBUTIONS

Malin Edqvist and Christine Rubertsson designed the study, with input from Cecilia Häggsgård, Pia Teleman, Gunilla Tegerstedt, and Gunilla Ajne. Christine Rubertsson was the principal investigator of the project. Malin Edqvist, Karin Ängeby, Cecilia Häggsgård, and Helena Tern were responsible for data collection related to the 1‐year follow‐up questionnaire. Data analysis was performed by Susann Ullén and Cecilia Häggsgård. Malin Edqvist and Cecilia Häggsgård drafted the initial version of the manuscript. All authors contributed to the interpretation of the results, critically revised the manuscript, and approved the final version.

## FUNDING INFORMATION

This study was funded by the Swedish Research Council for Health, Working Life and Welfare number 2018‐01192; Jan Hain's Foundation for Scientific Clinical Medical Research; and Skane County Council's Research and Development Foundation.

## CONFLICT OF INTEREST STATEMENT

The authors state explicitly that there are no conflicts of interest in connection with this article.

## ETHICS STATEMENT

The study was approved July 27, 2018, by the Regional Ethics Committee in Lund, Sweden, with the reference no. 2018–476. All women in the study had consented to participate in the trial. When consenting to participate in the trial, this included consenting to receiving follow‐up questionnaires at 1 month and 1 year postpartum. The Oneplus trial is registered at ClinicalTrials.gov, NCT03770962.

## Supporting information


**Table S1.** Description of data sources used in the study.
**Table S2.** Items used from the 1‐year follow‐up questionnaire including Pelvic Floor Impact Questionnaire (PFIQ‐7) and study‐specific items.
**Table S3.** Pelvic Floor Disability Index—20 (PFDI‐20) with reduced number of items, items used are marked in yellow.

## Data Availability

The data that support the findings of this study are available on request from the corresponding author. The data are not publicly available due to privacy or ethical restrictions.
