# Supplementary material for: Pelvic floor symptoms and quality of life 1 year postpartum in Swedish primiparous women—A follow‐up of a randomized controlled trial
Source: Acta Obstet Gynecol Scand. 2026 Apr 24;105(7):1275–86. doi: 10.1111/aogs.70216 (PMC13308974; doi:10.1111/aogs.70216)
Supplement: Supplementary file 1 — Table S1. Description of data sources used in the study. Table S2. Items used from the 1‐year follow‐up questionnaire including Pelvic Floor Impact Questionnaire (PFIQ‐7) and study‐specific items. Table S3. Pelvic Floor Disability Index—20 (PFDI‐20) with reduced number of items, items used are marked in yellow. [file AOGS-105-1275-s001.docx]

# Supporting Information

**Supporting information Table S1.** Description of data sources used in the study

| **Data from CRF** | **Data from medical database** | **Data from the one-year questionnaire** |
| --- | --- | --- |
| Ethnicity | Maternal chronic diseases | Marital status |
| Onset of the second stage (time) | Parity | Tobacco use |
| OASI | Onset of labour | BMI |
| Episiotomy | Mode of birth | Educational level |
| No tear | Time of birth | PFDI-20 |
| First-degree tear |  | PFIQ-7 |
| Minor second-degree tear | OASI |  |
| Major second-degree tear | Episiotomy |  |
| Rectal examination | Apgar score at 5 minutes |  |
| Tear assessed by two assessors | Birth weight |  |

| **Supporting information Table S2.** Items used from the one-year follow-up questionnaire including Pelvic Floor Impact Questionnaire (PFIQ-7) and study specific items   1. What is your highest level of education?   Primary School  Secondary School  University education 1-3 years  University education over 3 years  Other education, please describe _____________________________________  How much do you weigh today? _________kg  How tall are you? __________cm  Do you smoke?   - Yes - No   What is your current marital status?   - Single, please go to question **16** - Married/living with your partner - Have a partner but do not live together - Other, please describe: _____________________________________________________   **Questions regarding urinary incontinence**  Do you suffer from urinary incontinence?   - No, please go to question **65** - Yes - Sometimes     **UDI-6 (see below, Table S3)**  **PFIQ-7 urinary distress**  **Some women find that bladder symptoms affect their activities, relationships and feelings. Select the alternative that best describes how much your activities, relationships or feelings have been affected by your bladder symptoms or conditions during the past month.**  **How do symptoms or difficulties from your bladder (bladder control) affect your activities, relationships and emotions?**  My bladder control symptoms/difficulties affect my ability to do household chores (cooking, housecleaning, laundry)  □ Not at all □ Somewhat □ Moderately □ Quite a bit  My bladder control symptoms/difficulties affect my ability to do physical activities such as walking, swimming, or other exercise  □ Not at all □ Somewhat □ Moderately □ Quite a bit  My bladder control symptoms/difficulties affect my ability to attend entertainment activities such as going to a movie or concert  □ Not at all □ Somewhat □ Moderately □ Quite a bit  My bladder control symptoms/difficulties affect my ability to travel by car or bus for a distance greater than 30 minutes away from home  □ Not at all □ Somewhat □ Moderately □ Quite a bit  My bladder control symptoms/difficulties affect my ability to participate in social activities outside my home  □ Not at all □ Somewhat □ Moderately □ Quite a bit  My bladder control symptoms/difficulties affect my emotional health (e.g. nervousness, depression, etc)  □ Not at all □ Somewhat □ Moderately □ Quite a bit  My bladder control symptoms/difficulties lead to feelings of frustration/anger  □ Not at all □ Somewhat □ Moderately □ Quite a bit  **Additional:** My bladder control symptoms/difficulties lead to me having negative thoughts and feelings about my body  □ Not at all □ Somewhat □ Moderately □ Quite a bit  **Questions about flatus and faecal incontinence**  Do you have problems with involuntarily passing gas?   - No, please go to question 67 - Yes  1. How often do you have problems with involuntarily passing gas?   □ Almost never □ 1-3 times/month □ 1-3 times/week □ Daily   1. Do you have difficulties with faecal incontinence?  - No, please go to question **91** - Yes  1. If you answered *Yes* to question 65, how often do you have difficulties with faecal incontinence?   □ Almost never □ 1-3 times/month □ 1-3 times/week □ Daily   1. Do you use protection pads against faecal incontinence?  - No - Almost never - Yes, 1-3 times/month - Yes, 1-3 times/week - Yes, daily   **CRAD-8 (see below, Table S3)**  **PFIQ-7 related to colorectal-anal distress**  **Some women find that their bowel symptoms affect their activities, relationships and feelings. Select the alternative that best describes how much your activities, relationships or feelings have been affected by your bowel symptoms or conditions during the past month.**  **The following questions are about how symptoms or difficulties related to your bowel movements affect your activities, relationships and emotions.**  My bowel control symptoms/difficulties affect my ability to do household chores (cooking, housecleaning, laundry)  □ Not at all □ Somewhat □ Moderately □ Quite a bit  My bowel control symptoms/difficulties affect my ability to do physical activities such as walking, swimming, or other excersie  □ Not at all □ Somewhat □ Moderately □ Quite a bit  My bowel control symptoms/difficulties affect my possibilities to attend entertainment activities such as going to a movie or concert  □ Not at all □ Somewhat □ Moderately □ Quite a bit    My bowel control symptoms/difficulties affect my ability to travel by car or bus for a distance greater than 30 minutes away from home  □ Not at all □ Somewhat □ Moderately □ Quite a bit  My bowel control symptoms/difficulties affect my ability to participate in social activities outside my home  □ Not at all □ Somewhat □ Moderately □ Quite a bit  My bowel control symptoms/difficulties affect my emotional health (e.g. nervousness, depression, etc)  □ Not at all □ Somewhat □ Moderately □ Quite a bit  My bowel control symptoms/difficulties lead to feelings of frustration/anger  □ Not at all □ Somewhat □ Moderately □ Quite a bit  **Additional:** My bowel control symptoms/difficulties lead to me having negative thoughts and   feelings about my body  □ Not at all □ Somewhat □ Moderately □ Quite a bit  **POPDI-6 (see below, Table S3)**  **Some women think that vaginal symptoms affect their activities, relationships, and feelings. Select the alternative that best describes how much your activities, relationships or feelings have been affected by your vaginal symptoms or conditions during the past month**  The following questions are about how symptoms or difficulties related to your **vaginal** symptoms affect your activities, relationships and emotions.  My vaginal symptoms/difficulties affect my ability to do household chores (cooking, housecleaning, laundry)  □ Not at all □ Somewhat □ Moderately □ Quite a bit  My vaginal symptoms/difficulties affect my ability to do physical exercise such as walking, swimming, or other exercise:  □ Not at all □ Somewhat □ Moderately □ Quite a bit  My vaginal symptoms/difficulties affect my possibilities to attend entertainment activities such as going to a movie or concert  □ Not at all □ Somewhat □ Moderately □ Quite a bit  My vaginal symptoms/difficulties affect my ability to travel by car or bus for a distance greater than 30 minutes away from home  □ Not at all □ Somewhat □ Moderately □ Quite a bit  My vaginal symptoms/difficulties affect my ability to participate in social activities outside my home  □ Not at all □ Somewhat □ Moderately □ Quite a bit  My vaginal symptoms/difficulties affect my emotional health (e.g. nervousness, depression, etc)  □ Not at all □ Somewhat □ Moderately □ Quite a bit  My vaginal symptoms/difficulties lead to feelings of frustration/anger  □ Not at all □ Somewhat □ Moderately □ Quite a bit  **Additional:** My vaginal symptoms/difficulties lead to me having negative thoughts and feelings about my body:  □ Not at all □ Somewhat □ Moderately □ Quite a bit  **Additional items related to POP/vaginal symptoms**  Do you think that your vaginal opening is too wide/large?  □ No □ Yes If *yes*, how much does this bother you?  □ Not at all □ A little □ Quite a lot □ A lot  Do you think that your vaginal opening is too narrow/small?  □ No □ Yes If *yes*, how much does this bother you?  □ Not at all □ A little □ Quite a lot □ A lot |
| --- |

**Supporting information Table S3.** Pelvic Floor Disability Index – 20 (PFDI-20) with reduced number of items, items used are marked in yellow

| **PFDI-20** | **Item** | **Comment** |
| --- | --- | --- |
| **POPDI-6** | Do you usually experience pressure in the lower abdomen? | *The original subscale with all items included was used* |
|  | Do you usually experience heaviness or dullness in the pelvic area? |  |
|  | Du you usually have a bulge or something falling out that you can see or feel in your vaginal area? |  |
|  | Do you ever have to push on the vagina or around the rectum to have or complete a bowel movement? |  |
|  | Do you usually experience a feeling of incomplete bladder emptying? |  |
|  | Do you ever have to push up on a bulge in the vaginal area with your fingers to start or complete urination? |  |
|  | Response options  □ No □ Yes If *yes*, how much does this bother you?  □ Not at all □ A little □ Quite a lot □ A lot |  |
| **CRAD-8 -** modified with six items included | Do you feel you need to strain hard to have a bowel movement? | *Item included in the modified subscale* |
|  | Do you feel you have not completely emptied your bowels at the end of a bowel movement? | *Item included in the modified subscale* |
|  | Do you usually lose stool beyond your control if your stool is well formed? | *Item included in the modified subscale* |
|  | Do you usually lose stool beyond your control if your stool is loose? | *Item included in the modified subscale* |
|  | Do you usually have pain when you pass stool? | *Item* ***not*** *included in the modified subscale* |
|  | Do you usually lose gas from the rectum beyond your control? | *Item included in the modified subscale* |
|  | Do you experience a strong sense of urgency and have to rush to the bathroom to have a bowel movement? | *Item included in the modified subscale* |
|  | Does part of your bowel ever pass through your rectum and bulge outside during or after bowel movement? | *Item* ***not*** *included in the modified subscale* |
|  | Response options  □ No □ Yes If *yes*, how much does this bother you?  □ Not at all □ A little □ Quite a lot □ A lot |  |
| **UDI-6 -** modified with three items included | Do you usually experience frequent urination? | *Item* ***not*** *included in the modified subscale* |
|  | Do you usually experience urine leakage associated with a feeling of urgency, that is, a strong sensation of needing to go to the bathroom? | *Item included in the modified subscale* |
|  | Do you usually experience urine leakage related to coughing, sneezing or laughing? | *Item included in the modified subscale* |
|  | Do you usually experience small amounts of urine leakage (that is, drops)? | *Item included in the modified subscale* |
|  | Do you usually experience difficulty emptying your bladder? | *Item* ***not*** *included in the modified subscale* |
|  | Do you usually experience pain or discomfort in the lower abdomen or genital region? | *Item* ***not*** *included in the modified subscale* |
|  | Response options  □ No □ Yes If *yes*, how much does this bother you?  □ Not at all □ A little □ Quite a lot □ A lot |  |
